# Link Between Non-Invasive Intrapartum Interventions and Cardiotocography Patterns, Amniotic Fluid Color, and Immediate Neonatal Outcomes

**DOI:** 10.3390/healthcare14070888

**Published:** 2026-03-30

**Authors:** Nuria Garcia-Cuadrado, Ana Fernandez-Araque, Zoraida Verde, Maria Sainz-Gil, Carlos Durantez-Fernandez, Rosa M. Cardaba-Garcia, Veronica Velasco-Gonzalez

**Affiliations:** 1 Doctoral Program in Health Sciences, Faculty of Nursing, University of Valladolid, 47005 Valladolid, Spain; 2 Department of Nursing, Faculty of Health Sciences, University of Valladolid, Campus of Soria, 42003 Soria, Spain; 3 Research Group Pharmacogenetics, Cancer Genetics, Genetic Polymorphisms and Pharmacoepidemiology, University of Valladolid, 47005 Valladolid, Spain; 4 Department of Biochemistry and Molecular Biology and Physiology, Faculty of Health Sciences, University of Valladolid, Campus of Soria, 42003 Soria, Spain; 5 Department of Cell Biology, Genetics, Histology, and Pharmacology, Faculty of Medicine, University of Valladolid, 47005 Valladolid, Spain; 6 Department of Nursing, Faculty of Nursing, University of Valladolid, 47005 Valladolid, Spain; 7 Nursing Care Research (GICE), Faculty of Nursing, University of Valladolid, Av. Ramón y Cajal 7, 47005 Valladolid, Spain

**Keywords:** cardiotocography, labor, maternal repositioning, intrapartum management, neonatal outcomes

## Abstract

**Highlights:**

**What are the main findings?**

- Maternal positional changes are associated with suspicious or pathological intrapartum CTG.
- Meconium-stained amniotic fluid is more frequently observed in suspicious or pathological patterns but does not necessarily imply worse neonatal outcomes.

**What are the implications of the main findings?**

- Maternal repositioning is one of the most commonly used intrauterine resuscitation measures in cases of suspicious or pathological CTG patterns.
- The findings support the use of maternal postural changes in conjunction with other interventions depending on the clinical context, as well as the interpretation of CTG from a holistic perspective, taking into account maternal, fetal and obstetric factors.

**Abstract:**

**Background:** Non-invasive intrauterine resuscitation measures, such as maternal repositioning and intravenous fluid therapy, are used in the presence of suspicious or pathological cardiotocographic (CTG) patterns during labor. However, evidence regarding their link with CTG abnormalities, amniotic fluid color, and immediate neonatal outcomes is limited. **Objectives:** To analyze the link between maternal repositioning and intravenous fluid therapy and the occurrence of suspicious or pathological intrapartum CTG patterns, as well as their relationship with amniotic fluid color and immediate neonatal effects. **Methods:** An analytical, observational, prospective study was conducted in women in labor with continuous monitoring. Changes in maternal position, administration of intravenous fluid therapy, CTG patterns, amniotic fluid color, and immediate neonatal outcomes were analyzed. Links were evaluated using appropriate statistical tests, considering maternal positions in isolation and in combination. **Results:** Maternal repositioning, both alone and in combination, was associated with the presence of suspicious or pathological CTG and with statistically significant differences in the 5 min Apgar score when analyzed as a continuous variable. No significant association was observed between intravenous fluid therapy and CTG patterns or neonatal outcomes. The presence of meconium-stained amniotic fluid was associated with a higher frequency of suspicious or pathological CTG. **Conclusions:** Maternal repositioning was most frequently applied as a clinical response to a suspicious CTG. Intravenous fluid therapy showed no link with CTG abnormalities or adverse neonatal outcomes. These findings reinforce the need to interpret intrapartum CTG in an integrated manner with the overall clinical context and support the use of maternal repositioning as a non-invasive measure in intrapartum management.

## 1. Introduction

The monitoring of fetal well-being during labor aims to identify fetal compromise early on, thereby guiding obstetric clinical management and optimal perinatal outcomes. Cardiotocography (CTG) is the most widely used non-invasive test for assessing fetal well-being during labor. However, despite its widespread use, it has high sensitivity but low specificity for predicting adverse neonatal outcomes, which can lead to unnecessary interventions and significant variability among different obstetricians regarding the subjectivity of the criteria used for CTG interpretation and decision-making [1,2,3].

International guidelines have been published by the International Federation of Gynecology and Obstetrics (FIGO) [4,5] and the American College of Obstetricians and Gynecologists (ACOG) [6]. These organizations establish standardized criteria for classifying CTG patterns obtained by CTG as normal, suspicious, and pathological by systematically evaluating the basal fetal heart rate, the variability of that rate, and the presence of accelerations and decelerations from a baseline. However, available evidence indicates that the presence of a non-reassuring or pathological CTG does not always result in adverse neonatal outcomes [4,5,7]. This poses significant challenges for decision-making during labor, which in many cases is carried out without the certainty provided by the diagnostic test itself [3].

When abnormal cardiotocographic patterns are detected, various non-invasive interventions aimed at improving fetal oxygenation and uteroplacental perfusion are recommended before considering other invasive measures [6,8,9,10]. 

Among these interventions, one of the most common is repositioning the mother, as it is simple, well accepted by the pregnant woman and involves no cost. Repositioning the mother is an easy, well-accepted and free intervention designed to help reduce maternal aortocaval compression or transient umbilical compression [9,11,12]. Furthermore, the administration of intravenous fluid therapy using crystalloid solutions has been used by other authors with the aim of improving maternal hemodynamic status and uteroplacental blood flow, although the available evidence regarding its impact on CTG changes and neonatal outcomes remain limited and heterogeneous [5,13,14,15,16,17]. Overall, the scientific literature suggests that a combination of various interventions may be beneficial for perinatal outcomes [4,5,15,16].

On the other hand, certain intrapartum factors, such as amniotic fluid color, have been associated with the development of non-reassuring cardiotocographic patterns. In particular, meconium-stained amniotic fluid during the dilation process has been linked to an increased risk of CTG abnormalities and a potential fetal compromise. However, its clinical interpretation must always be considered in conjunction with other fetal and obstetric parameters, as current trends indicate that the fetus’s digestive maturity may cause the amniotic fluid to become stained by the passage of meconium; this does not necessarily imply a poor neonatal prognosis, but may simply be a consequence of the fetus’s own maturity [5,13,18].

The assessment of the immediate neonatal condition after birth is usually performed by scoring the Apgar test at the first and fifth minutes of life. The scores achieved in the test are to be widely used as indicators in clinical practice and observational studies to assess early neonatal adaptation [19]. This test provides a rapid and reproducible assessment of the newborn’s well-being in the first minutes of life by evaluating motor, respiratory, cardiovascular, and skin coloring parameters [19,20]. Various intrapartum clinical practices, as well as maternal and obstetric factors, may influence neonatal outcomes, highlighting the need to systematically analyze the relationship between care interventions during delivery, CTG findings and neonatal outcome [5,7,19], which in turn may be influenced by other factors that have been under-researched to date.

Therefore, the objective of this study was to analyze the link between maternal position and the administration of intravenous fluid therapy during childbirth, with the occurrence of non-reassuring or pathological CTG in term pregnant women during labor. It also sought to determine the relationship between amniotic fluid color and the presence of abnormal CTG. There is a possible relationship between the Apgar score at one and five minutes, in newborns, and the use of maternal repositioning and the administration of intravenous fluids during labor.

## 2. Materials and Methods

### 2.1. Study Design

This is an analytical, observational, prospective study based on the collection of variables through fieldwork carried out by the researcher herself and collaborators trained for this purpose.

### 2.2. Target Population, Sample, Sample Size and Sampling Technique

The target population for the study consisted of women giving birth at the University Clinical Hospital of Valladolid (Spain). These comprised approximately 950 women who give birth at this hospital on average each year; women giving birth at the Medina del Campo Regional Hospital in Valladolid (Spain), numbering approximately 250 women who give birth at this hospital each year; and women giving birth at the Río Hortega University Hospital in Valladolid (Spain), which is approximately 1560 women who give birth at this hospital each year. The total population was approximately 2760 women. In all cases, these are public hospitals.

The required sample size was calculated using the QuestionPro^®^ tool (https://questionpro.com, updated 24 April 2024), based on a population of 2760 women in labor, with a 95% confidence level and a 5% margin of error. The estimated sample size was 338 women; this figure was exceeded, indicating that the sample can be considered representative of the population.

The study included full-term pregnant women in labor with a single fetus in cephalic presentation and continuous monitoring, during the dilation phase, from a central region of Spain (Valladolid, Castilla y León).

Not all pregnant women who were initially selected for the study were able to take part, despite meeting the inclusion criteria. Some were excluded for various reasons: (1) the development, during the dilation phase, of irreversible complications or the presence of cardiotocographic signs indicating the need for immediate delivery of the fetus, usually by cesarean section; or (2) failure to sign the informed consent form.

The sampling technique used was non-probabilistic convenience sampling based on the care provided to women by the principal investigator and the collaborators trained in data collection, that is to say, consecutive.

The sample size was calculated using the QuestionPro^®^ tool for a population of 2760 women in labor, with a confidence level of 95% and a margin of error of 5%. The estimated simple size was 338 women; however, this number was significantly exceeded.

The participant recruitment period ran from 1 November 2021 to 31 December 2022.

### 2.3. Data Collection Procedure

A research team was formed comprising a network of midwives with specific training in CTG interpretation in accordance with FIGO classification criteria, in order to avoid bias in the interpretation of the trace and to ensure consistent data collection. Three face-to-face group training sessions and one online session specific to each participating hospital were held.

Participants were recruited by the research team during the pre-delivery fetal well-being scan between 38 and 40 weeks’ gestation, where they received verbal information about the study and provided their written informed consent prior to inclusion. They were able to ask questions during that session, but data collection began with the active phase of labor.

During labor, care was provided by a member of the research team. The standard clinical variables associated with the labor process were systematically recorded on the partogram (the document used to record labor). In addition, changes in the mother’s position, the administration of intravenous crystalloid solutions, and any event potentially associated with the emergence of a suspicious or pathological pattern were recorded on the cardiotocographic chart and noted in the document.

Subsequently, in order to minimize potential bias and avoid subjective interpretations when compiling the database, the principal investigator accessed the clinical records of each participant, which included a review of both documents used to record the variables. The primary variable was analyzed using the pair review method, initially classified by the research team during labor and subsequently reviewed by the principal investigator. This procedure helped to reduce inter-observer variability and enhance the reliability of the data obtained. Intra-observer variability had already been addressed during the training of the research team by the principal investigator.

### 2.4. Study Variables

#### 2.4.1. Main Variable

The main variable of the study was the intrapartum CTG, classified as normal, suspicious, or pathological according to FIGO criteria [4,5], and dichotomized as the presence or absence of risk of fetal distress (RLFW). RLFW was defined as the presence of a suspicious or pathological cardiotocographic trace, in accordance with the criteria of the Spanish Society of Gynaecology and Obstetrics (SEGO) [21].

As mentioned earlier, the FIGO guidelines for intrapartum CTG are classified into three categories: normal, suspicious or abnormal [4]. It is important to clarify that, for the purposes of this study, the presence of ‘risk of fetal distress’ (RLFW) was not based solely on the cardiotocographic pattern. This categorization was a composite variable that integrated the presence of suspicious or pathological CTG patterns together with the occurrence of predefined intrapartum clinical events or risk factors (such as uterine hyperdynamics, maternal fever, or hypotension, as listed in Table 1). Therefore, the ‘RLFW’ category represents a clinical construct of non-reassuring intrapartum status, rather than an isolated CTG pattern classification.

#### 2.4.2. Secondary Variables

The following were considered secondary variables:

Sociodemographic and obstetric variables including maternal age, parity, and gestational age at birth.

Maternal position during labor, recorded as a categorical variable according to the position adopted (right lateral decubitus, left lateral decubitus, supine decubitus, prone decubitus and sitting), and analyzed in isolation (change in position: yes/no) and in combination, defined as the adoption of more than one position throughout labor. This variable was recorded systematically during labor, including cases in which positional changes were indicated by midwives in response to alterations in CTG. With regard to changing the mother’s position, regardless of whether the fetus was in a left or right position, the left lateral position was always chosen initially, as this is the position that improves uteroplacental perfusion [22]. If the cardiotocographic trace did not deteriorate, she was left in that position for at least 30 min. If, after this time, the trace improved, the woman was placed in a semifowler’s position and, if necessary, repositioned to the left lateral decubitus position for at least 30 min. If the trace did not improve in these positions, the right lateral decubitus position was adopted for at least 30 min. If the trace did not improve, other positions were tried and further therapeutic measures were taken; however, if it did improve, the semifowler’s position was attempted again.

Administration of intravenous serum therapy, specifying the type of serum administered, and analyzed in isolation (serum yes/no) and in combination; considered combined when different types of serum were administered during labor. Some authors believe that fluid therapy could improve placental blood flow and thereby reduce the risk of fetal distress. The fluids used are crystalloids, with 0.9% saline solution being the fluid of choice. Only 500 mL of intravenous Ringer’s lactate solution was administered prior to epidural analgesia to prevent maternal hypotension and, consequently, the risk of fetal distress. During labor, normal saline was routinely administered for maintenance, but in the event of a suspicious or abnormal fetal heart rate, a 500 mL bolus of this solution was administered. If the fetal heart rate did not improve within 10 min of completing the administration, the dose was repeated with a further 500 mL bolus. Once 1000 mL had been administered, no further bolus saline was given, but maintenance doses were continued (500 mL over 12 h) [23]. The use of 5% glucose solution is restricted to cases of maternal hypoglycemia or diabetes during labor [24].

Amniotic fluid color: Amniotic fluid is traditionally classified into three grades: grade 1 (mild) meconium is diluted by a large volume of amniotic fluid that is slightly stained with meconium, grade 2 meconium (moderate) is a reasonable amount of amniotic fluid with a heavy suspension of meconium, and grade 3 meconium (thick meconium) is present in little amniotic fluid, suggesting the presence of meconium in scant amounts of amniotic fluid. The assessment of the degree to which the amniotic fluid is stained is usually described using the symbols + for grade 1, ++ for grade 2 and +++ for grade 3 [25,26]. Amniotic fluid classification was performed according to the density and visual appearance of the meconium [25]. In this respect, peer review is more difficult to carry out, but the classification is widely used and standardized amongst obstetricians in Spain.

Clinical factors potentially associated with a non-reassuring or abnormal CTG, including uterine hyperactivity, maternal fever, maternal pushing, uterine hypertonicity with an external cardiotocographic focus, precipitous labor as defined by ICD-10 (lasting less than three hours), amniotomy, intrapartum blood loss, and unknown cause [27].

Regarding uterine hyperdynamia or tachysystole, the FIGO definition is used, which states that this refers to an excessive frequency of contractions and is defined as the occurrence of more than 5 contractions in 10 min, either in two successive 10 min periods or as an average over a 30 min period [28].

Maternal fever or hyperthermia is defined as a temperature of ≥38.0 °C on a single occasion or ≥37.5 °C on two occasions, at least one hour apart, in accordance with the criteria set out by Maude et al. In such cases, the woman is treated with 1 g of intravenous paracetamol every 8 h until the hyperthermia subsides, as recommended by SEGO. Temperature is measured using an OMRON Eco Temp Basic^®^ digital axillary thermometer (OMRON, Kyoto, Japan) [29].

Apgar score of the newborn, assessed at the first and fifth minutes of life, analyzed as a quantitative variable (mean ± SD) and categorical variable (<7/≥7). This score was obtained according to standard clinical parameters (color, heart rate, muscle tone, reflex response, and respiration), following the classic Apgar description and its clinical use in neonatology [30,31]. For quantitative analyses, the score was used as a continuous variable (mean ± SD). For categorical analyses, the Apgar classification <7 and ≥7 was used, based on widely accepted clinical criteria that consider scores ≥ 7 to be reassuring and scores < 7 to be indicative of a higher probability of neonatal compromise or the need for additional intervention [30,31].

### 2.5. Statistical Analysis

Descriptive statistical analysis was performed using absolute frequencies and percentages for qualitative variables and means and standard deviations for quantitative variables. Pearson’s chi-square test was used to analyze the relationship between two qualitative variables. Ultimately, linear and logistic regression analyses were performed, resulting in a stepwise binomial logistic regression model based on the Wald test.

The IBM SPSS (Statistical Package for Social Science; IBM, Armonk, NY, USA) v.29.0 statistical package, licensed by the University of Valladolid, was used to create the database. The statistical significance value was the usual ≤0.05.

### 2.6. Ethical Considerations

The study was approved by the Drug Research Ethics Committees (CEIm) of the two Health Areas in which it was carried out: the Valladolid East Health Area, through the CEIm of the Valladolid University Clinical Hospital (reference PI 21-2343 THESIS), and the Valladolid West Health Area, through the CEIm of the Río Hortega University Hospital (reference 21-PI20). The processing and use of data were carried out in accordance with current legislation on personal data protection in Spain and the European Union, including the Organic Law on Personal Data Protection and Guarantees of Digital Rights 3/2018 [31], Regulation (EU) 2016/679 of the European Parliament and of the Council of 27 April 2016 (GDPR) [32], as well as the principles established in the Declaration of Helsinki [33]. Confidentiality and anonymity of the subjects were preserved by using three-digit numerical codes and omitting personal details of the cases included in the audits.

The quality of the study was ensured by following the STROBE Guide of the EQUATOR strategy for observational studies [34].

## 3. Results

### 3.1. Descriptive Results of the Study Sample

**Figure 1:**
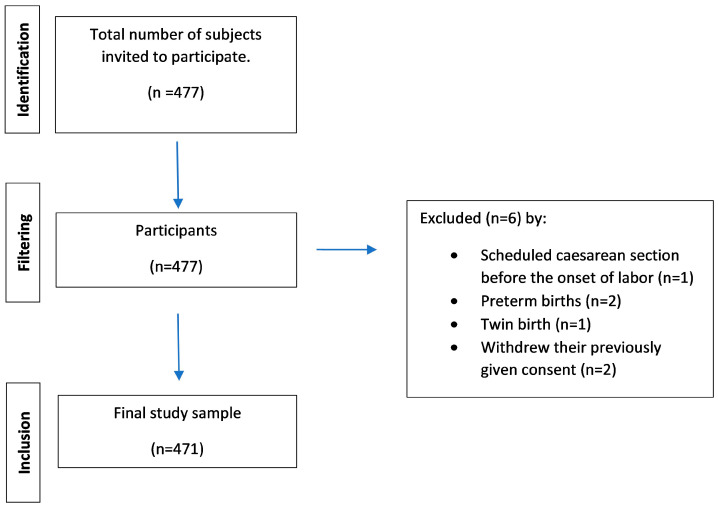
Participant flow diagram.

The initial sample consisted of 477 women, of whom complete and valid records were obtained for 471 (98.74%), after excluding one woman who had a scheduled cesarean section before the onset of labor, two who had preterm births, one who had a twin birth, and two who withdrew their previously given consent (Figure 1).

Regarding the sociodemographic data collected for this sample of parturient women, the average age was 33.37 years (±5.86). Delivery occurred on average at 39.69 weeks (±1.25). Regarding the parity of the women at the time of the study, the average was 1.54 children (±0.77).

Of the data collected on newborns, the Apgar scores at one minute and five minutes are noteworthy. In the first case, the average was 8.68 points and in the second case, 9.58 points.

The rest of the variables related to the delivery process that are most relevant to the objectives of the study are presented in Table 1.

### 3.2. Results of Relational Analyses Between Two Study Variables

Following the descriptive analysis, significant links were sought between two variables that were part of the study. This was the case of factors associated with a non-reassuring or pathological CTG and different maternal positions during labor. Similarly, links were sought between non-reassuring or pathological CTG and the use of different types of intravenous therapy during labor. Cross-tabulation tables were used to facilitate the calculation of the Chi-square statistic.

A statistically significant link was observed between maternal positions, both used alone and in combination, and the distribution of possible mediating factors in obtaining a non-reassuring or pathological CTG (*p* = 0.013; Cramér’s V = 0.417). The exact results can be found in Table 2. The factor most frequently associated with CTG alterations in both the group of women in labor who were repositioned and those who were not repositioned was of unknown origin. It should be noted that in several of these cases the subsample is less than five women, indicating that caution should be exercised with this result.

In contrast, no statistically significant differences were found between the factors associated with a non-reassuring or pathological CTG and the administration of intravenous fluid therapy alone or in combination according to the Chi-square test (*p* = 0.775).

A relational analysis was also performed using cross-tabulation to calculate the Chi-square statistic between the color of the amniotic fluid and the presence of a non-reassuring or pathological CTG, obtaining a statistically significant link between the variables indicated (*p* < 0.05; Cramér’s V = 0.261). In several cases, the subsample was less than five, so these results should also be interpreted with caution (Table 3).

The link between the color of the amniotic fluid and changes in maternal positions was analyzed both individually and in combination using contingency or cross tables and the Chi-square test. No relationship was observed between the color of the amniotic fluid and the maternal positions adopted during the delivery process, considered jointly (*p* = 0.41). Similarly, the link between amniotic fluid color and the administration of intravenous fluids was analyzed in isolation and in combination using the Chi-square test, with no statistically significant link found in these cases (*p* = 0.883). The Apgar score at 1 min and 5 min, expressed as a categorical variable according to values <7 and ≥7, was compared according to the isolated maternal position and the isolated administration of serum therapy. No statistically significant links were observed between the Apgar score at 1 min and maternal position alone (*p* = 0.175) or with serum therapy alone (*p* = 0.884). Similarly, the Apgar score at 5 min showed no significant differences based on the non-combined position (*p* = 0.293) or the single administration of fluids (*p* = 0.293).

When the Apgar score was analyzed as a quantitative variable, differences were observed depending on the isolated maternal position. There was a statistically significant relationship between changing the position of women in labor in isolation during delivery and the mean Apgar scores at 1 min (*p* = 0.049) and 5 min (*p* = 0.002) (Table 4).

With regard to the administration of isolated intravenous fluids, no statistically significant differences were observed in the mean Apgar scores at either 1 min (*p* = 0.690) or 5 min (*p* = 0.973).

### 3.3. Results of Regression Analysis and Predictive Model Generation

Finally, linear regression and logistic regression analyses were performed to identify possible predictors of the Apgar score at 1 min and 5 min, taking into account the dependent variable in the quantitative and qualitative formats. Initially, the variables of maternal position change during delivery, either alone or in combination, administration of serum therapy alone or in combination, amniotic fluid color and factors associated with a non-reassuring or pathological CTG were considered in isolation without obtaining regression models. Subsequently, the sociodemographic variable of maternal age and those related to obstetric aspects (gestational age at birth and parity) were incorporated. In this case, a logistic regression model was found by applying the Wald method for the newborn’s Apgar score at 5 min in qualitative format, in which a constant and the mother’s parity (β = 0.99) intervene. This model has limited predictive power according to Cox and Snell’s R^2^ index (0.074 = 7.4%) and Nagelkerke’s R^2^ index (0.136 = 13.6%). Table 5 below shows all the data from the regression analysis.

## 4. Discussion

Based on the results obtained, the main objective of this study, to examine the link between certain non-invasive intrapartum clinical interventions, particularly maternal position and the administration of intravenous fluid therapy, and the occurrence of suspected or pathological intrapartum CTG, has been achieved. In addition, it was possible to determine whether there was a relationship with the color of the amniotic fluid and immediate neonatal effects. The main findings showed that maternal position, both in isolation and in combination, was associated with the presence of abnormal CTGs and with statistically significant differences in the 5 min Apgar score when analyzed as a continuous variable, whereas no significant link was observed between intravenous fluid therapy and CTGS patterns or neonatal outcomes. Likewise, the presence of meconium-stained amniotic fluid was linked with a higher frequency of suspicious or pathological CTG.

The proportion of suspicious or pathological CTGs detected in women in labor, which affected approximately 25% of pregnant women, is within the range of variability described by the results of other studies [4,11,35]. Both these previous studies and international clinical practice guidelines indicate that the frequency of non-reassuring tracings depends largely on the characteristics of the obstetric population, the care protocols of the hospital, and the classification systems used [4,5,6]. In hospital settings with continuous intrapartum monitoring and protocolized care, as in this study, it is common for a significant proportion of tracings to be classified as suspicious without this necessarily translating into adverse neonatal outcomes [6,14,35], reinforcing the need to interpret CTG in an integrated manner with the rest of the intrapartum clinical picture and not as an isolated finding [4,6,35,36]. In any case, the results must be viewed in the context of social factors, as by Brezeanu et al. [37].

About maternal position, the results of this research suggest that isolated or combined postural changes during labor may be associated with the appearance of abnormal CTG. This link probably reflects the clinical indication for maternal repositioning in response to the detection of an alarming CTG, rather than a direct causal effect of the intervention itself. This finding should be interpreted with caution because despite the fact that previous studies indicate that certain changes in maternal position, especially the left lateral decubitus position, may improve uteroplacental perfusion by reducing aortocaval compression [10,12,15,35,38], in our study, postural changes were primarily performed as a clinical response to the presence of altered CTG. Therefore, the link observed does not imply a beneficial effect of repositioning per se, but could reflect that postural changes applied in isolation or without specific selection of the most effective position are not always sufficient to normalize the tracing, especially when the altered CTG is due to other pathophysiological mechanisms, such as umbilical cord compression [14,36], where combined positions could have a greater benefit [15]. This could suggest a reverse causality, which would need to be verified by further studies. However, given the observational design of the study, it is not possible to establish a causal relationship between the maternal position and the onset or resolution of CTG alterations. Furthermore, a considerable proportion of the altered tracings had an unidentified etiology, reinforcing the multifactorial nature of intrapartum cardiotocographic alterations and the need for an integrated clinical interpretation that considers maternal, fetal and obstetric variables, such as maternal age, gestational age, parity, obstetric history, course of pregnancy, presence of pregnancy-related pathology, amniotic fluid color, and duration of labor, among others [4,6,14,35,36]. CTG remains the most widely used tool for identifying RLFW during labor and for guiding clinical decision-making [4,6,35]. However, numerous authors have pointed out its high sensitivity and limited specificity, particularly in suspicious tracings, as well as interobserver variability in its interpretation [4,6,14,35,36]. Suspicious cardiotocographic patterns may reflect various pathophysiological mechanisms, such as decreased uteroplacental perfusion, umbilical cord compression, tachysystole or uterine hypertonia, maternal arterial hypotension, intrapartum infection, or fetal response to stress during the expulsive period [4,6,14,35]. The high percentage of cases with unidentified causes in this study is consistent with the previous research and underscores the importance of interpreting CTG in context, integrating maternal, fetal, and obstetric information [4,6,14,35,36].

With regard to intravenous serum therapy alone or in combination with isotonic (Ringer’s lactate and 0.9% saline) and hypotonic (5% glucose) crystalloids, this study found no significant link with the onset of RLFW or immediate neonatal outcome determined by the Apgar score. This finding is consistent with the available literature, which shows heterogeneous results and limited evidence on the clinical benefit of maternal intravenous hydration as an intrauterine resuscitation measure during labor [15,16,17,39]. It is also true that assessing neonatal adaptation solely on the basis of the Apgar test is limited, and that other parameters should be used alongside this test to complement it [40].

While fluid therapy has been suggested as a means of improving maternal hemodynamic status and uteroplacental flow, frequently in conjunction with postural interventions, in the context of RLFW the existing evidence is insufficient to confirm a clear impact on cardiotocographic patterns or neonatal outcomes, thereby supporting the need for additional research [4,17]. The results of this study support this position and suggest that, in low-risk populations and in protocolized care settings, the efficacy of serum therapy in modifying CTG patterns or improving fetal and neonatal outcomes remains uncertain [15,17] and dependent on clinical indication and care context [39], which could explain the absence of a link observed in this predominantly low-risk cohort.

The presence of meconium-stained amniotic fluid was significantly associated with the type of CTG, with a higher frequency observed in suspected or pathological CTGs [13,25]. In the scientific literature, the interpretation of meconium-stained amniotic fluid remains controversial. On the one hand, it has been described as being associated with a higher frequency of CTG abnormalities, acidosis, or the need for intrapartum intervention, especially when the meconium is thick and coexists with other signs of fetal compromise [41]. On the other hand, pathophysiological reviews suggest that, in term pregnancies, meconium expulsion may reflect gastrointestinal and vagal maturation, without necessarily requiring RLFW, which would explain why some newborns with meconium-stained fluid show normal neonatal adaptation [17,18]. In this study, the link between meconium-stained amniotic fluid and a higher proportion of suspicious or pathological CTGs supports its usefulness as an intrapartum marker of the need for increased surveillance or alertness, although it does not in itself allow us to infer a worse neonatal prognosis [4,42], especially when the 5 min Apgar score remains within reassuring ranges. In any case, this design does not allow us to establish cause-and-effect relationships, only correlations. This finding is consistent with previous studies describing a higher frequency of cardiotocographic abnormalities in this low-risk population with continuous intrapartum monitoring [4,14,35]. However, and in line with the recommendations of international clinical practice guidelines during the delivery process [4,6,10], meconium should always be interpreted in conjunction with other clinical parameters as a marker requiring closer monitoring, and not as an isolated indicator of fetal compromise [6,8,9,10,36]. This study found a relationship between the presence of meconium-stained fluid and pathological or non-reassuring CTGs, suggesting that the theory of meconium elimination as part of fetal maturity does not hold true in the study sample, especially considering that these were full-term deliveries. In these women, the presence of meconium-stained amniotic fluid warrants close monitoring by healthcare professionals attending labor [5,25,42].

With regard to neonatal outcomes, no significant differences were observed in Apgar scores at one minute and five minutes when analyzed as a categorical variable (<7/≥7). However, when considering the Apgar score as a continuous variable with scores from 0 to 10 points, statistically significant differences were identified related to the maternal position defined in isolation and without a combination of positions. Although these differences were small and of limited clinical relevance, their detection is of interest from an epidemiological perspective and supports the use of the 5 min Apgar score as an indicator of early neonatal adaptation in observational studies, whilst always taking into account its clinical relevance in combination with other neonatal parameters [30,43,44]. These results were consistent with the literature that recognizes the value of the Apgar test for immediate neonatal assessment, albeit with limitations in establishing specific etiological diagnoses [30,31,40,45].

In the multivariate analysis, parity remained associated with the five-minute Apgar score when the test was analyzed as a continuous variable, with slightly lower values observed in newborns based on maternal parity. It is true that the significance of this finding is limited and that its clinical relevance is not particularly great either, but this finding is consistent with previous studies that have described a potentially slower initial neonatal adaptation in the context of first-time births [46,47], possibly related to longer labor, increased obstetric interventions or an altered physiological response to intrapartum stress [47,48]. However, the predictive capacity of the model was limited, indicating that parity alone can explain only a small proportion of the variability in the newborn’s Apgar score at five minutes, highlighting the multifactorial nature of immediate neonatal adaptation and the need to incorporate other variables that have not been assessed in this study, thereby enabling the development of more robust regression models. Furthermore, this link was not observed when the Apgar score was analyzed categorically or in the continuous score at one minute, suggesting a small effect of limited clinical relevance, which, as mentioned, could be improved through further research.

Despite the evidence described by other authors, the absence of a link between other intrapartum variables and neonatal outcomes can be explained by several factors. Firstly, the study population consisted mainly of low-risk pregnant women, whose newborns had generally high Apgar scores, which limits the variability of the outcomes and reduces the probability of detecting clinically relevant differences [49,50]. Secondly, the small size of some subsamples, such as the group that received intravenous fluid therapy, reduces statistical power. Finally, although the Apgar test is a widely used tool for assessing early neonatal adaptation, it is not a direct marker of fetal hypoxia or acidosis [19,44]; the absence of biochemical parameters such as pH or lactate in the fetus limited the pathophysiological interpretation of the CTG abnormalities, and this may contribute to discrepancies with studies that use more objective measurements [35].

This study has certain limitations that should be taken into account when interpreting the results. Its design precludes the establishment of causal relationships, and there was a loss of participants, although this was not found to be high, as well as measurement bias, for example regarding the color of the amniotic fluid. The non-probabilistic sampling and the small size of some subsamples limit the generalizability of the findings. Furthermore, the absence of direct biochemical markers of fetal distress restricts the pathophysiological interpretation of the observed cardiotocographic patterns. It should also be borne in mind that there may be a reverse causality bias related to changes in maternal position and the CTG. Regarding the classification of cardiotocographic tracings, this study opted to dichotomize them. This decision was methodologically driven by our primary objective, which focused on the presence of intrapartum clinical events and risk factors (such as maternal fever, hypotension, or uterine hyperdynamics) that inherently place the maternal–fetal dyad in a clinically ‘non-reassuring’ category, irrespective of whether the resulting tracing met FIGO criteria for ‘suspicious’ or ‘pathological’.

Overall, the results of this study provide observational evidence in real-world conditions on the relationship between non-invasive intrapartum interventions, CTG findings, and immediate neonatal outcomes based on the Apgar score. The results support the use of maternal repositioning as an initial measure in the management of RLFW, while underscoring the need for further research into its clinical impact through studies with more robust methodological designs. Furthermore, they reinforce the importance of a comprehensive approach to intrauterine fetal resuscitation, in which clinical decision-making is based on an overall assessment of the cardiotocographic trace and the maternal and fetal context [4,6,14,35,36]. Given the exploratory nature of some subgroup analyses, particularly those presented in Table 2 and Table 3 which contain cells with small counts (n < 5), a post hoc power analysis was considered. However, due to the small size of these specific subgroups, the statistical power for detecting significant differences in those particular comparisons is limited. Consequently, these specific results must be interpreted with caution, as they are presented for descriptive purposes and hypothesis generation, and are not intended to support definitive conclusions.

Future studies with controlled trials could help to clarify the combined effect of these interventions and identify subgroups of pregnant women who may benefit more clearly from certain intrapartum management strategies. It would also be interesting to further investigate the clinical significance of meconium-stained amniotic fluid, analyzing its relationship with gestational age and cardiotocographic patterns, to discern whether its presence is due to a physiological phenomenon of fetal maturation or to intrapartum compromise.

## 5. Conclusions

This observational study provides evidence in real-world conditions on the relationship between certain non-invasive intrapartum interventions, CTG findings, and immediate neonatal outcomes. Maternal repositioning was linked with the presence of non-reassuring or pathological CTG patterns and with statistically significant, albeit clinically insignificant, differences in Apgar scores analyzed as a continuous variable, whereas intravenous fluid therapy was not linked with CTG patterns or neonatal outcomes. Similarly, the presence of meconium-stained amniotic fluid was associated with a higher frequency of abnormal CTG, reinforcing its role as an intrapartum clinical marker requiring closer monitoring, regardless of its possible physiological origin. Taken together, these findings support a comprehensive, contextualized approach to intrapartum fetal monitoring, in which clinical decisions are guided by the overall interpretation of the cardiotocographic trace in the context of maternal and fetal factors, rather than by isolated finding. Future prospective studies with more robust and methodological designs are needed to clarify the clinical impact of these interventions and identify subgroups of pregnant women who may benefit from specific intrapartum management strategies.

## Figures and Tables

**Table 1:** Description of the main study variables.

Variable	n (%)
*Color of amniotic fluid* (n = 471)	
Clear	379 (80.5)
Meconium +/+++	44 (9.3)
Meconium ++/+++	36 (7.6)
Meconium +++/+++	10 (2.1)
Hematic	2 (0.4)
*Suspected RLFW* (n = 471)	
Yes	133 (28.2)
No	338 (71.8)
*Possible factors related to RLFW due to non-calmed or pathological CTG* (n = 133)	
Uterine hyperdynamic	2 (1.5)
Maternal arterial hypotension	3 (2.3)
Maternal pushing	17 (12.8)
Maternal fever	14 (10.5)
Uterine rupture	4 (3.0)
Amniotomy	2 (1.5)
Uterine hypertonia	2 (1.5)
Precipitate labor	5 (3.8)
Unknown cause	74 (55.6)
Bleeding	10 (7.5)
*Maternal repositioning during delivery* (n = 175)	
RLD	67 (38.3)
LLD	78 (44.6)
D semifowler (SD)	19 (10.9)
PD	2 (1.1)
Seated at 90°	9 (5.1)
*Serum therapy administered during delivery* (n = 36)	
0.9% saline solution	2 (5.6)
Ringer’s lactate solution	16 (44.4)
5% glucose solution	18 (50.0)

The meconium-stained amniotic fluid scale is measured from a minimum of one symbol + to a maximum of three symbols + [21,22]; RLFW: risk of fetal distress; RLD: right lateral decubitus; LLD: left lateral decubitus; SD: supine decubitus; PD: prone decubitus.

**Table 2:** Relationship between factors associated with the presence of non-reassuring or pathological CTG according to the change in maternal position adopted during the delivery process.

Factors Associated with Non-Reassuring or Pathological CTG	Change in Maternal Position During Labor n (%)	No Change in Maternal Position During Labor n (%)	*p* Value
Hyperdynamic	0 (0.0)	2 (2.1)	0.013
Pushing	5 (20.0)	12 (12.6)	
Maternal fever	4 (16.0)	10 (10.5)	
Maternal arterial hypotension	2 (8.0)	1 (1.1)	
Uterine rupture	2 (8.0)	2 (2.1)	
Hypertonia	0 (0.0)	2 (2.1)	
Precipitate labor	3 (12.0)	2 (2.1)	
Amniotomy	0 (0.0)	1 (1.1)	
Amniotomy (p. precipitate)	1 (4.0)	0 (0.0)	
Unknown	8 (32.0)	63 (66.3)	

CTG: cardiotocographic recording, p. precipitate: precipitate delivery.

**Table 3:** Relationship between amniotic fluid color and the presence of non-reassuring or pathological CTG.

Color of Amniotic Fluid	Normal CTG n (%)	Suspicious/Pathological CTG n (%)	*p* Value
Clear	295 (87.5)	86 (65.4)	<0.05
Meconium +	26 (7.5)	25(14.6)	
Meconium ++	15 (4.5)	22 (16.2)	
Meconium +++	1 (0.3)	1 (0.8)	
Hematic	1 (0.3)	3 (2.3)	
Total	338	133	

The meconium-stained amniotic fluid scale is measured from a minimum of one symbol + to a maximum of three symbols + [25,26].

**Table 4:** Apgar score as a quantitative variable according to change in maternal position.

Apgar Test	No Change in Position n (Mean ± SD)	With Change in Position n (Mean ± SD)	*p* Value
Apgar at 1 min	35 (8.66 ± 0.97)	97 (8.19 ± 1.69)	0.049
Apgar at 5 min	35 (9.89 ± 0.40)	97 (9.46 ± 1.12)	0.002

Total n = 132 due to missing data in one participant; SD: standard deviation.

**Table 5:** Forward stepwise logistic regression analysis (dependent variable: Apgar score at 5 min as a categorical variable).

Variables	β	*p*	OR	CI 95%
Intersection	3.76	0.048		
Parity	0.99	0.001	0.77	0.34–1.49

OR: odds ratio, CI: confidence interval.

## Data Availability

The data presented in this study are available upon request from the corresponding author due to privacy restrictions.

## Abbreviations

The following abbreviations are used in this manuscript:

CTG	Cardiotocographic recording
FIGO	International Federation of Obstetrics and Gynecology
CEIm	Committee on Ethical Review of Drug Trials
RLFW	Risk of loss of fetal well-being
SEGO	Spanish Society of Gynaecology and Obstetrics
ICD	International Classification of Diseases
